# Genetic Background and Allorecognition Phenotype in *Hydractinia symbiolongicarpus*

**DOI:** 10.1534/g3.111.001149

**Published:** 2011-11-01

**Authors:** Anahid E. Powell, Maria Moreno, Andrea Gloria-Soria, Fadi G. Lakkis, Stephen L. Dellaporta, Leo W. Buss

**Affiliations:** * Department of Ecology and Evolutionary Biology; † Department of Molecular, Cellular, and Developmental Biology, and; ‡ Department of Geology and Geophysics, Yale University, New Haven, Connecticut 06511; § Thomas E. Starzl Transplantation Institute, Pittsburgh, Pennsylvania 15261, and; ** Department of Surgery; †† Department of Immunology; ‡‡ Department of Medicine, University of Pittsburgh School of Medicine, Pittsburgh, Pennsylvania 15261

**Keywords:** hydroid, cnidarian, invertebrate immunity, recognition

## Abstract

The *Hydractinia* allorecognition complex (ARC) was initially identified as a single chromosomal interval using inbred and congenic lines. The production of defined lines necessarily homogenizes genetic background and thus may be expected to obscure the effects of unlinked allorecognition loci should they exist. Here, we report the results of crosses in which inbred lines were out-crossed to wild-type animals in an attempt to identify dominant, codominant, or incompletely dominant modifiers of allorecognition. A claim for the existence of modifiers unlinked to ARC was rejected for three different genetic backgrounds. Estimates of the genetic map distance of ARC in two wild-type haplotypes differed markedly from one another and from that measured in congenic lines. These results suggest that additional allodeterminants exist in the *Hydractinia* ARC.

Colonial marine invertebrates often encounter conspecifics when encrusting hard surfaces in the sea. As such animals have limited or no capacity for movement, cell-to-cell contact between individuals inevitably results. Allorecognition at such contacts triggers a sequence of events that typically involves a binary choice between fusion and rejection.

Allorecognition decisions have long been appreciated to be under genetic control based on the observation that fusion is rare except among close kin. A more detailed genetic understanding of fusibility is available for two organisms, both of which have developed into model systems for the study of this phenomenon. The two systems are *Botryllus schlosseri*, an invertebrate chordate, and *Hydractinia symbiolongicarpus*, a cnidarian and the subject of this study [recently reviewed by Rosengarten and Nicotra (2011)].

The genetic analysis of fusibility in *Hydractinia* began with the work of Hauenschild (1954). In a series of crosses with wild-type animals he provided support for a one-locus system with alleles of different strength. Hauenschild’s scheme was largely successful in accounting for the segregation patterns he observed, but several fusibility results defied explanation based on a one-locus model. Dupasquier (1974) reanalyzed Hauenschild’s data and noted that, if one assumed the existence of two linked loci and further assumed that one of Hauenschild’s crosses involved a recombinant animal, then several (but not all) of the original inconsistencies were resolved.

No further work was reported for another 22 years until Mokady and Buss (1996) developed a near-isogenic line. Their study was based on a large number of crosses each with a small segregating population. Fusibility was shown to segregate as a single chromosomal interval within the line. The development of a near-isogenic line was followed by the development of a near-congenic line. Using the congenic line, Cadavid *et al.* (2004) mapped the chromosomal interval and showed that it comprised at least distinct two loci. The interval was called ARC (for allorecognition complex), and the allorecognition loci were designated *alr1* and *alr2*.

The allorecognition loci *alr1* and *alr2* have recently been identified by positional cloning (Nicotra *et al.* 2009; Rosa *et al.* 2010). Both genes encode putative transmembrane receptor proteins with extracellular domains resembling Ig-like domains and with intracellular sequence motifs similar to immunoregulatory signaling motifs. Both proteins bear hypervariable domains most similar to Ig-like V-set domains. Comparison of several wild-type alleles of both loci identified multiple V-set residues under positive selection. Notably, *alr1* was found to be contained within a large family of structurally similar immunoglobulin superfamily-like genes (Rosa *et al.* 2010).

Knowledge of the *alr1* and *alr2* genotype fully predicted fusibility within the inbred and congenic lines (Cadavid *et al.* 2004; Powell *et al.* 2007). Specifically, if animals shared one ARC haplotype, they fuse, and if they share no ARC haplotype, they reject. Animals that do not share an allele at one *alr* locus but do share an allele at the other *alr* locus undergo a form of transitory fusion, where colonies initially fuse and thereafter separate from one another. The chronology and other aspects of the phenomenology of the separation vary depending on the locus at which the alleles are shared (Powell *et al.* 2007).

Whether the “fusion rules” elucidated for the congenic lines were sufficient to explain wild-type variation in fusibility remained an open question until the *alr* loci were identified. Fusion is rare in the field (Nicotra and Buss 2005). Two separate tests of fusibility in wild-type colonies have been performed. In the first, wild-type animals were found that fused or underwent transitory fusion with animals from inbred lines, and the sequence of *alr* alleles was obtained to determine if they matched. A survey of 535 animals yielded two such animals and both bore matching alleles (Nicotra *et al.* 2009; Rosa *et al.* 2010). In a related test, wild-type *alr1* and *alr2* alleles were sequenced, and fusion tests were subsequently performed on colonies found to bear one or more matching alleles. Five pairs of colonies were identified as bearing matching alleles, and four of the five pairs displayed a fusible phenotype (Rosa *et al.* 2010). In both tests, transitory fusion was observed in some colonies, when permanent fusion was expected (or vice versa).

These observations indicate that *alr1* and *alr2* are major determinants of allorecognition in the wild, but they also indicate that additional allodeterminants are yet to be identified. Two alternative genetic explanations are readily suggested. These additional allodeterminants could be unlinked modifying loci that were heretofore undetected because they were homogenized by the inbreeding program employed to identify the ARC. Alternatively, unlinked modifying loci may play a minimal role, but additional, unidentified loci within different ARC haplotypes may act as allodeterminants. Under this hypothesis, such loci differ between wild-types and inbred haplotypes but not within the congenic lines.

The first of these hypotheses can be easily addressed by classical breeding approaches. The conventional test for dominant modifiers is to introduce alleles from an inbred line into a wild-type genetic background and test for the appearance of the predicted phenotype in homozygotes derived in backcross or F2 incross progeny (Green 1981). We provide such a test for three different genetic backgrounds and report no effect of genetic background on fusibility. Unexpectedly, these crosses yielded different map distances for the size of the ARC complex from that repeatedly measured for congenic lines.

## Methods

### Nomenclature, genetic lines, and wild-type animals

ARC haplotypes are labeled by letter, so a homozygous animal is designated as ARC-f/f and a heterozygous animal as, say, ARC-f/r. We make use of two near-inbred lines and one near-congenic line. These two inbred lines are designated as ARC-f/f and ARC-r/r. The congenic line has the ARC-r haplotype introgressed into the ARC-f/f genetic background. The complete pedigree of the lines can be obtained by concatenating previously published pedigrees (Cadavid *et al.* 2004; Mokady and Buss 1996; Poudyal *et al.* 2007; Powell *et al.* 2007).

We tested the effect of genetic background by using three wild-type animals. OQ6D, with haplotypes designated as ARC-c and ARC-d, was collected from the shallow subtidal just seaward of One Tree Island in Old Quarry Harbor, Guilford, CT, in 2002. LH06-082, with haplotypes ARC-a and ARC-b, was collected intertidally from Lighthouse Point, New Haven, CT, in 2006. LH06-003, with haplotypes ARC-i and ARC-r2, were collected intertidally in 2006 at Meig’s Point, Madison, CT. No ARC alleles are shared between the three wild-types, indicating that the three wild-types are not closely related.

The sequences of both *alr1* and *alr2* for each haplotype have been published previously (Nicotra *et al.* 2009; Rosa *et al.* 2010; Rosengarten *et al.* 2011). Two of the three genetic backgrounds were chosen because the original wild-type animals were identified as giving a phenotypic response different from that expected based on fusion rules developed for congenic animals. Specifically, LH06-082 bears an ARC-f–like haplotype and displays transitory fusion when paired with an ARC-f/f tester, when fusion would be expected (Nicotra *et al.* 2009; Rosa *et al.* 2010). LH06-003 has an *alr2*-r–like allele and would be predicted to display transitory fusion to an ARC-r/r tester, but instead, it rejects. Note that using such backgrounds increases the likelihood that we will detect unlinked modifiers if they exist.

### Crosses and fusibility testing

**Figure 1:**
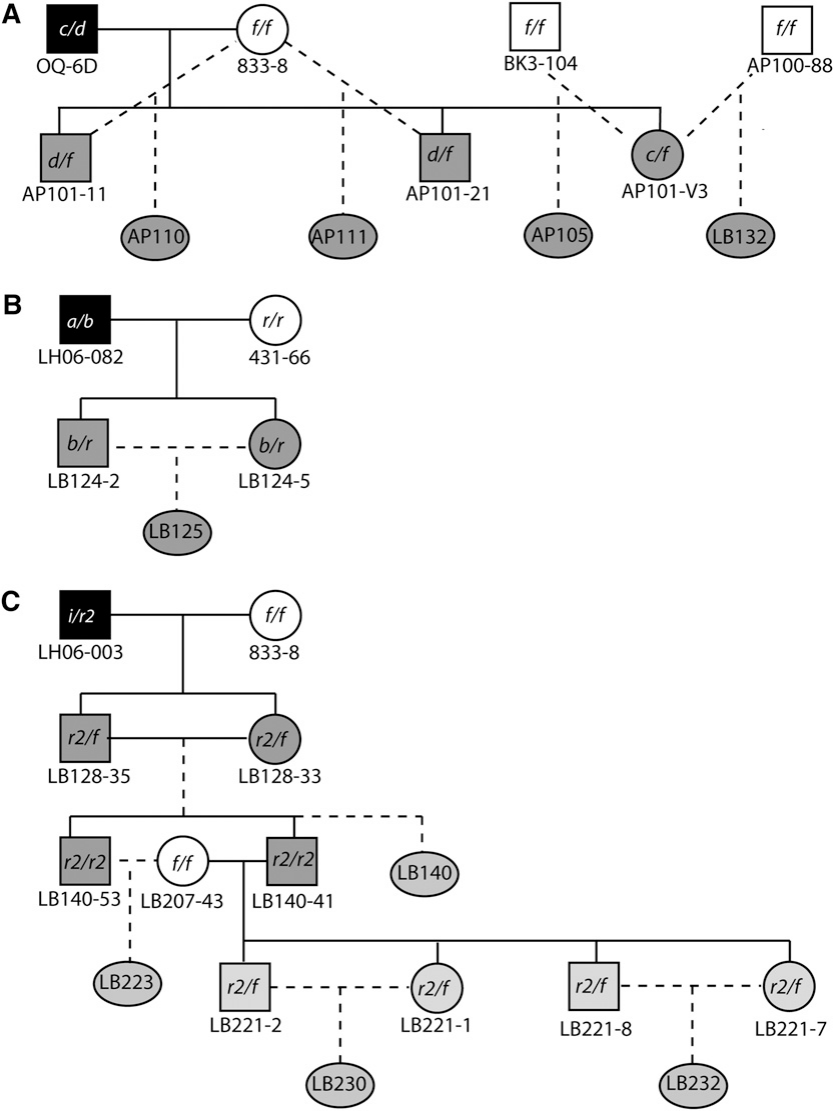
Pedigree of crosses used to generate mapping populations. Males are represented by boxes, females by circles. Black represents individuals collected from the wild, white represents laboratory inbred lines, and lighter and darker shades of gray represent 50% and 25% contributions of the wild-type genetic background, respectively. ARC haplotypes are given inside icons, and colony identification numbers are underneath. Backcrosses and sib crosses used to generate homozygous ARC haplotypes in 50% or 25% wild-type background are shown as dashed lines, and the resulting progeny population identification numbers are shown in ellipses. Lineage derived from wild-type (A) OQ6D, (B) LH06-082, and (C) LH06-003.

Crosses were made using established techniques (Cadavid *et al.* 2004; Mokady and Buss 1996; Powell *et al.* 2007), and colonies were maintained under standard conditions (Blackstone and Buss 1991). To test for the presence of unlinked dominant, codominant, or incompletely dominant allorecognition modifiers, we crossed a wild-type animal with an inbred or cogenic line and retrieved progeny homozygous for inbred ARC haplotypes. Inbred colonies used for matings are given in supporting information, Table S2, and Figure 1. In the OQ6D background we retrieved ARC homozygotes from either backcrosses (*i.e.* crosses AP100 and AP101 in Figure 1A) or crosses to an inbred colony that was near-isogenic to the original inbred parent (*i.e.* crosses AP105 and LB132 in Figure 1A). The latter were necessary to include female progeny crossed with male inbreds and are effectively identical to backcrosses, as all inbred animals are near isogenic. In the LH06-082 and LH06-003 genetic backgrounds, we used sib crosses to retrieve ARC homozygous progeny (Figure 1, B and C). In LH06-003 background only two homozygous progeny were recovered from the initial F2 population, requiring a series of additional crosses (Figure 1C). Identifying homozygotes bearing inbred ARC haplotypes required that we genotype at two or more ARC molecular markers (see below) which yielded information on the frequency of recombination.

Progeny identified as homozygous for the inbred ARC-f and ARC-r haplotype were tested for fusibility against allocompatible inbred lines. Colony fusion assays were performed in the conventional manner (Buss *et al.* 1984; Powell *et al.* 2007). An ARC-f/f animal in a wild-type genetic background would be expected to fuse to an ARC-f/f inbred tester if there were no effective allorecognition modifier in the background. Any departure from the fusion phenotype is evidence for a modifier. An inbred ARC-f/f tester (colony IDs 833-8 and LB217-11, respectively) was used for tests involving the OQ6D and LH06-003 genetic backgrounds. Two ARC-r/r testers, one from an inbred line (colony ID 4117-2) and the other from the congenic line (colony ID MP104-34) were used for tests involving the LH06-082 background. Fusion tests were observed 3–7 days/week during the first two weeks and weekly thereafter for a minimum of three additional weeks, up to a maximum of three months.

Backcrosses and F2 incross progeny share 50% wild-type background and 50% inbred background, which is equivalent to saying that each individual had a 0.5 chance of inheriting a modifier. Each progeny provides an independent sample of the wild-type genetic background, so the number of individuals (*n*) that must be tested to ensure detection at a given probability is (0.5)*^n^* for a dominant modifier. An *n* > 5 is required for *P* < 0.05. The terminal populations in the LH06-003 pedigree (Figure 1C) have a 25% chance of inheriting a modifier. If *n* progeny are recovered from the F2 incross and *m* from the terminal populations, the probability of failing to detect a modifier is *P* = (0.75)^2^*^n^*^+^*^m^*.

### Molecular markers and mapping

Single polyp and larval DNA extractions were performed using the procedures described in Powell *et al.* (2007). Crosses in the OQ6D and LH06-082 genetic backgrounds were genotyped using markers that spanned the ARC to detect animals that were recombinant over the complex. Sample sizes precluded determination of map distances for LH06-003 and LH06-082 haplotypes.

With two exceptions, the markers used for ARC genotyping have been described earlier; all alleles, primers, and references are provided in Table S1. Conditions for the two new markers, 194c28 and 174i1, are as follows. Marker 194c28 was amplified in a 25 μl PCR containing 1 × Phusion HF Buffer, 3% DMSO, 0.4 μM each primer, 0.2 mM dNTP, and 0.5 μl of Phusion DNA polymerase. Reaction conditions were 98° for 30 s followed by 35 cycles of 98° 10s, 62° 30s, 72° 1 min, and a final extension at 72° 5 min. Products were digested with *Nde*I and analyzed on a 2% agarose gel. Marker 174i1 was amplified in a 25 μl PCR containing 10% PCR buffer (Qiagen, Valencia, CA), 0.2 mM dNTP, 0.8 unit of Taq DNA polymerase, 0.75 μM of common primer (1^st^ primer, Table S1) and 0.5 μM of each allele-specific primer (2^nd^ primer, Table S1). Reactions conditions were 94° for 1 min, 25 cycles of 94° 20s, 58° 30s, 72° 60s, with a final extension at 72° for 4 min.

**Figure 2:**
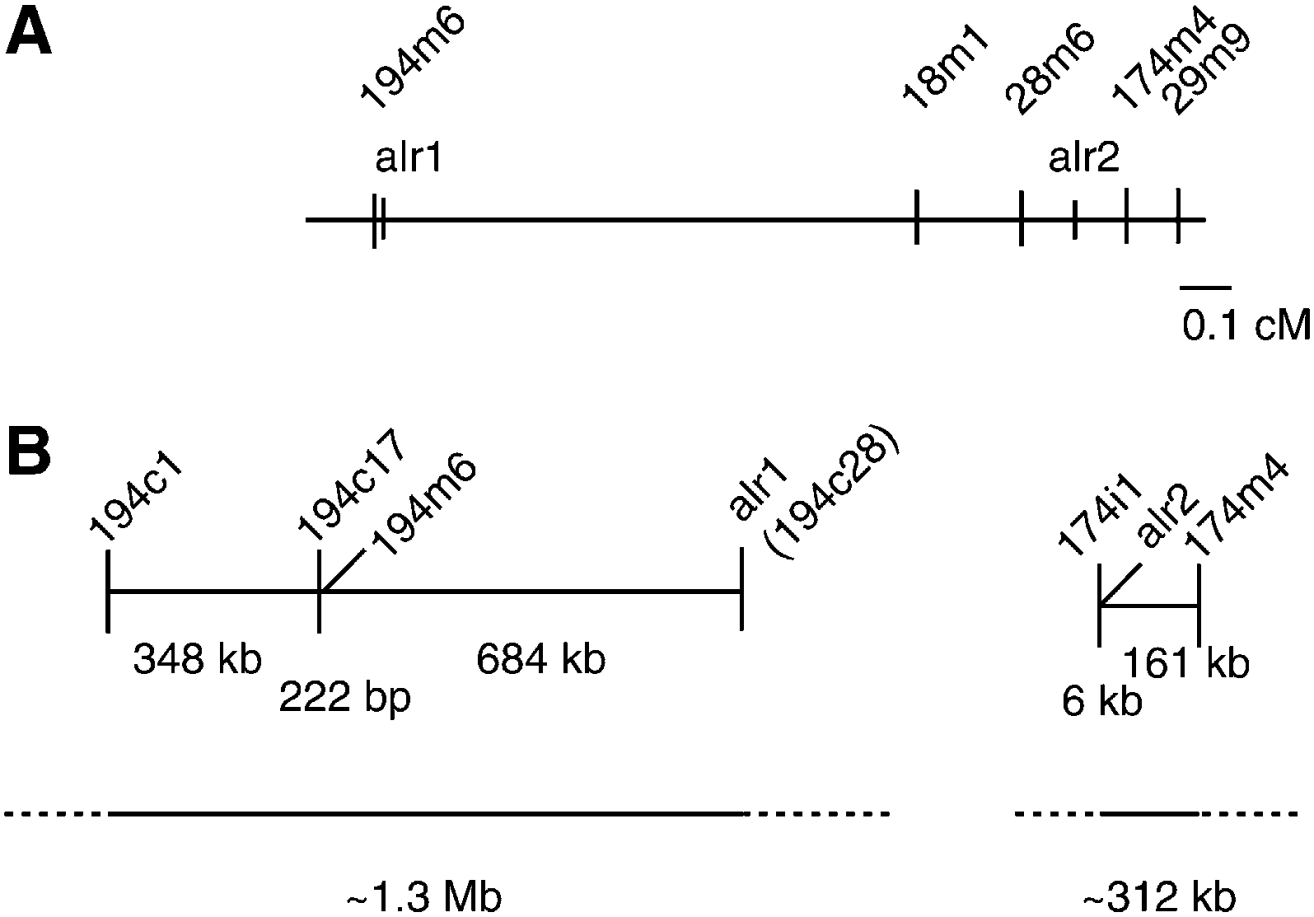
Locations of markers within the ARC. (A) Genetic map of the ARC from Powell *et al.* (2007) with genetic distance shown in centimorgans. (B) Relative spacing of markers on the both the 194 and 174 loci shown to scale. Contigs containing *alr*1 and *alr*2 of 1.3 Mb and ∼312 kb, respectively, have been sequenced. A third sequenced contig (not shown) of 0.7 Mb lies between these. The physical size of the intervening regions is not yet known.

The relative locations of the markers within the ARC are shown in Figure 2. Note that the ARC interval has not been physically closed, but the physical interval characterized spans in excess of 2.3 Mb. Markers designated with the same numerical prefix (*e.g.*194 or 174) are closely linked relative to the size of the interval. Hereafter, when we refer to the 194-174 distance in centimorgans, we treat markers with the same prefix as identical.

Map distances were computed using MapMaker (Lander *et al.* 1987). MapDraw (Liu and Meng 2003) was used to draw linkage maps to scale. To augment sample sizes, we repeated several crosses and extracted DNA from mature planulae. We pooled samples of larvae and colonies when testing segregation ratios and calculating map distances.

## Results

The crosses used to test for dominant modifiers in three different genetic backgrounds are presented in Table S2 and Figure 1. In the OQ6D genetic background, four crosses were made and homozygotes identified. A total of 37 ARC-f/f homozygous individuals were tested, and all exhibited permanent fusion to an ARC-f/f tester colony. In the LH06-082 genetic background, a single F2 incross was performed, and 10 different ARC-r/r homozygotes were tested. All progeny exhibited permanent fusion to the ARC-r/r tester. In the LH06-003 background, 2 ARC-f/f homozygotes were identified in an F2 population and an additional 8 homozygotes were identified from the terminal populations. All exhibited permanent fusion to the ARC-f/f tester. The hypothesis of dominant, codominant, or incompletely dominant modifiers of allorecognition assorting independently of ARC can be rejected for the OQ6D and LH06-082 backgrounds at *P* < 0.001 and rejected for the LH06-003 genetic background at *P* < 0.05.

Genotype data from the two ARC-f/f × ARC-f/d crosses were pooled. The two ARC-f/f × ARC-f/c crosses were genotyped using different markers and are reported separately. All markers for both crosses segregated in the expected single locus Mendelian ratios (Table 1). Raw data are available in Table S3.

**Table 1:** Marker segregation ratios

Cross	Marker	Genotype		N	χ^2^	*P*
AP110+AP111		f/f	f/d			
	194m6	266	225	491	3.42	0.064
	18m1	293	297	590	0.027	0.87
	28m6	299	298	597	0.0017	0.97
	174m4	299	291	590	0.108	0.74
AP105		f/f	f/c			
	194m6	126	101	227	2.75	0.097
	18m1	156	129	285	2.56	0.11
	28m6	156	129	285	2.56	0.11
	174m4	154	129	283	2.20	0.14
	29m9	153	126	279	2.61	0.11
LB132		f/f	f/c			
	194c17	35	32	67	0.13	0.72
	174	32	38	70	0.51	0.48

**Figure 3:**
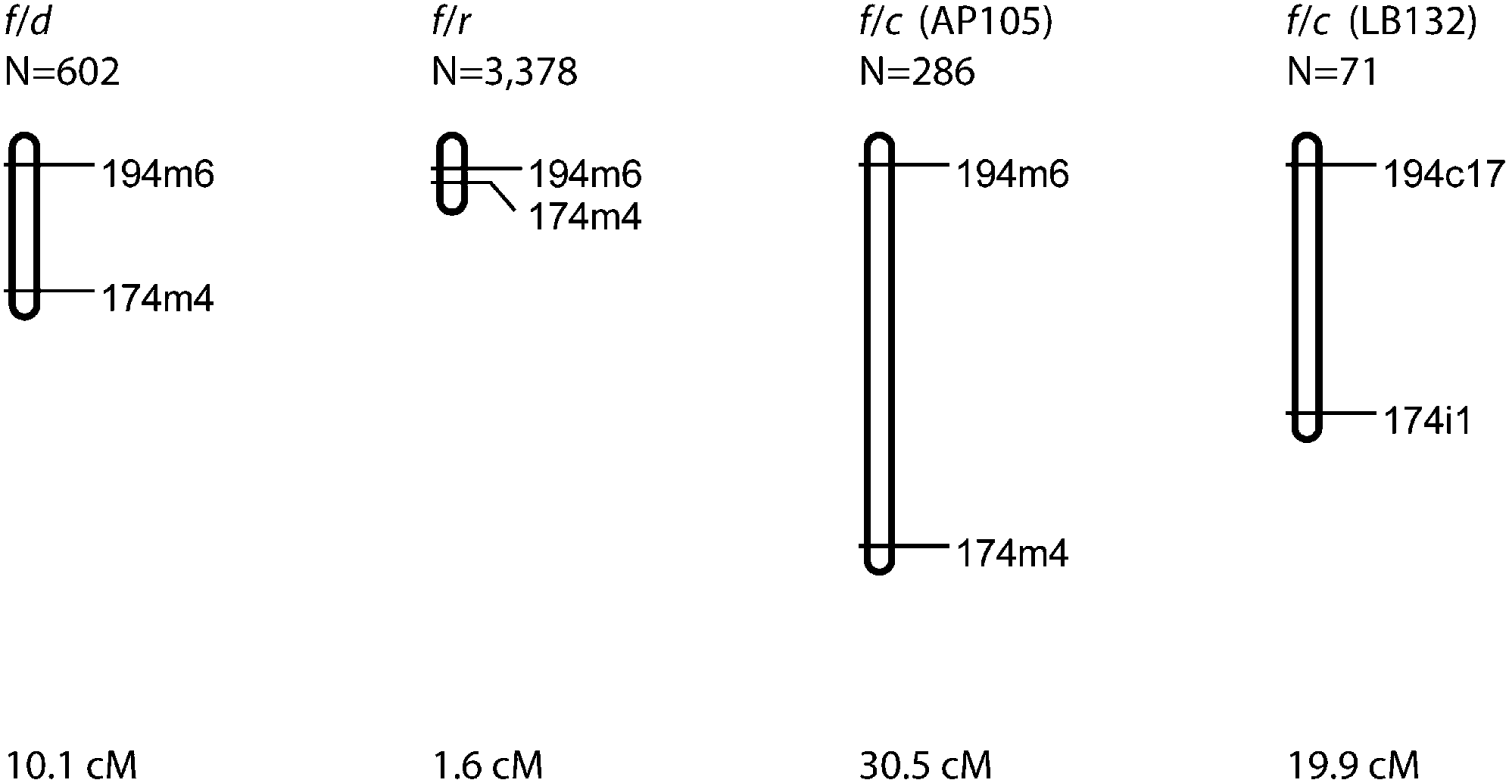
Genetic maps in centimorgans drawn to scale. The total map distance is given below each map. Data for the f/r map from Cadavid *et al.* (2004) and Powell *et al.* (2007).

Map distances between ARC genetic markers varied widely (Figure 3), ranging from 1.6 to 30.5 cM for the interval between markers 194 and 174. Two of these maps measured distances generated from f and c haplotypes. In the ARC-f/f × ARC-f/c cross, the order of markers 28m6 and 174m4 was inverted relative to that determined for the ARC-f/f × ARC-f/r cross and the ARC-f/f × ARC-f/d cross. The inverted map order was not well supported, with a relative log likelihood of −1.74, which corresponds to support by an odds ratio of 50:1. Both wild-type haplotype map distances were substantially in excess of the 1.6 cM previously measured using the congenic ARC-f/f × ARC-f/r crosses (Powell *et al.* 2007). Pairwise differences in map distances were evaluated using the total heterogeneity of fit statistic (Liu 1998). Whereas no significant heterogeneity was detected between the maps resulting from the two different *f/c* crosses (*P* = 0.08, total heterogeneity goodness of fit statistic), when these were compared to the *f/d* map, significant heterogeneity was detected (*P* < 0.01) The heterogeneity was not due to segregation distortion and can therefore be attributed to differences in the relative rates of recombination between haplotypes.

## Discussion

We failed to detect evidence for the assortment of dominant, codominant, or incompletely dominant modifiers of allorecognition in *Hydractinia symbiolongicarpus*. Previously, a small number of cases were reported in which the genotype of the two known allodeterminants (*alr1* and *alr2*) failed to account for wild-type fusibility (Rosa *et al.* 2010). Our results show that these cases cannot readily be explained by appeal to dominant modifying loci unlinked to the ARC. The results reported here, when combined with the recent finding that the ARC complex includes a large number of Ig-superfamily genes bearing structural similarity to the known *alr* loci, raises the distinct possibility that additional genes within the ARC complex may act as allodeterminants.

Two caveats are germane. First, the breeding designs (OQ6D background) or sample sizes (LH06-003 and LH06-082 backgrounds) used do not allow us to test for recessive modifiers of allorecognition. Second, modifiers might act in a dosage-dependent fashion. In this study, the entire ARC interval was introduced into a wild-type background. Modifiers might have been detected if the number of matching *alr* loci within the ARC differed between test and out-crossed animals. Both possibilities are amenable to further study by classical breeding techniques.

Unexpectedly, we found very substantial variation in map distances in two wild-type ARC haplotypes relative to the size reported for our congenic line. Our congenic line, involving the ARC-f and ARC-r haplotypes, has been measured in a number of crosses to be about 2 cM (Cadavid *et al.* 2004; Powell *et al.* 2007). The two ARC haplotypes examined here exceeded this figure by large factors. Recent findings bear note in this regard. First, the ARC is now known to be composed of a large number of structurally similar genes (Rosa *et al.* 2010). Although the full extent of the family is not yet known, the minimal size is 13 Ig-superfamily genes for ARC-f (Nicotra *et al.* 2009; Rosa *et al.* 2010). Tandemly duplicated gene families are known to be prone to both enhanced rates of recombination and gene conversion (Chen *et al.* 2007; Gangloff *et al.* 1996; Norman *et al.* 2009; Robbins *et al.* 1991). Indeed, patterns of sequence similarity between wild-type *alr1* alleles and other ARC loci strongly suggest that different loci within the ARC complex may serve as sequence donors to one another (Rosa *et al.* 2010). Such processes may be expected to lead to structural divergence between haplotypes. A study of the genomic interval surrounding *alr*2 in three different haplotypes has documented extensive structural variation (Rosengarten *et al.* 2011). The large map distances detected in wild-type haplotypes raise the possibility that more extensive recombination between *alr1* and *alr2* is the norm. A high frequency of recombination may be expected to obscure single locus segregation when testing fusibility between progeny populations involving wild-type animals as was once reported (Grosberg *et al.* 1996).The small map distance documented in the congenic line might, for example, be generated by structural variation between haplotypes. Resolution of this issue will require the assembly of a reference sequence of the full ARC complex and subsequent comparison of the interval from multiple haplotypes.

## Supplementary Material

Supporting Information
