# Supplementary material for: Genetic Background and Allorecognition Phenotype in Hydractinia symbiolongicarpus
Source: G3 (Bethesda). 2011 Nov 1;1(6):499–504. doi: 10.1534/g3.111.001149 (PMC3276163; doi:10.1534/g3.111.001149)
Supplement: Supporting Information [file supp_1_6_499__index.html]

Supporting Information 

# Genetic Background and Allorecognition Phenotype in *Hydractinia symbiolongicarpus*

## Supporting Infomation for Powell *et al.*, 2011

**Files in this Data Supplement:**

- Supporting Information - Table S1-S3 (PDF, 328 KB)
- Table S1 - ARC Molecular Markers (PDF, 60 KB)
- Table S2 - Crosses (PDF, 60 KB)
- Table S3 - Raw data (PDF, 60 KB)
