# Supplementary material for: Genetic Background and Allorecognition Phenotype in Hydractinia symbiolongicarpus
Source: G3 (Bethesda). 2011 Nov 1;1(6):499–504. doi: 10.1534/g3.111.001149 (PMC3276163; doi:10.1534/g3.111.001149)
Supplement: Supporting Information [file supp_1.6.499_TableS1.pdf]

Table S1 ARC Molecular Markers

Marker ID	1st Primer	2nd Primer	Amplicon Length	Extension Primer	Polymorphic haplotypes	Reference
Sequenom Markers						
194m6	GACATACATAATTTACGTAAC	CCATTGATTTTAGGACGTGAC	120	GGACGTGACTTAACAGA	*f, c, d*	1
18m1	TGCAGCAAATGGTGATGTAC	ACAGACGAAATGGGAAATCC	114	CCATGTTGTAAAATACGCC	*f, c, d*	1
28m6	AGCGACGGGCTTAAGGTTTT	CGAAGTTCTTGATACACATGC	112	TTGTTAATTTTCTCACTCGTAA	*f, c, d*	1
174m4	AAATATTCAAACTGATGCTC	AGCCGAAACAGTTATCAGTC	115	AATTTTTTATTGTGCGGAAC	*f, c, d*	1
29m9	GAATTCATTTTTCTAAACAG	TGCATTGGATTGAAGCAAAG	107	GATTGAAGCAAAGAAGTTTAG	*f, b, c, d*	1
CAPS Markers			ARC-ff size (bp)	Enzyme	Digest size (bp)	
194c17	TCGCTGTTAAGAAGCCTAAAAGA	ATATTAGCAATGCGAAGGAGGGTGA	940	αTaq1	*f*: 657/281<br>*r*: 481/284/180<br>*c*: ~250	2
194c1	ATTAGTTCAGTTCAGGCCAAGA	CCTTCGATACCTCTACGATGAC	741	MaeIII	*f, b*: 60, 148, 533<br>*r*: 208, 533	3
194c28	TCATCTGGTGGTGGTGAAC	TAYCGATCCAACYCGTTCA	363	NdeI	*f*: 363<br>*r2:* 197,166	4
PCR indel markers						
174i1	TTGGATGATTCCTGCAAACGA	CCAGTTAGCTGAATAAAGCTTTGGA	284 (*f* allele)			4
		TGCTCATTATTTATCAGCTATTCATTCA	171 (*r, r2* allele)			4
SNP polymorphisms detected by sequencing						
alr2 exon9	TGACGGAACTTACGACCAACTCGAA	AACAAGAAAATGGTGCCGCTCTTTG	531	Amplified sequence contains 7 SNPs identified by sequencing		5

1 (Powell *et al.* 2007)

2 (Powell 2008)

3 (Rosa *et al.* 2010)

4 Conditions provided in text.

5 (NICOTRA 2007)
