# Supplementary material for: Genetic Background and Allorecognition Phenotype in Hydractinia symbiolongicarpus
Source: G3 (Bethesda). 2011 Nov 1;1(6):499–504. doi: 10.1534/g3.111.001149 (PMC3276163; doi:10.1534/g3.111.001149)
Supplement: Supporting Information [file supp_1.6.499_TableS2.pdf]

**Table S2 Crosses**

| Background | Cross ID | Female | Genotype | Male | Genotype | Size of mapping population | | |
|---|---|---|---|---|---|---|---|---|
| | | | | | | Larvae | Colony | Total |
| OQ6D | AP101 | 833-8 | ARC-ff | OQ6D | ARC-cd | | | |
| | AP110 | 833-8 | ARC-ff | AP101-11 | ARC-df | 90 | 50 | 140 |
| | AP111 | 833-8 | ARC-ff | AP101-21 | ARC-df | 451 | 11 | 462 |
| | AP105 | AP101-V3 | ARC-cf | BK3-104 | ARC-ff | 231 | 55 | 286 |
| | LB132 | AP101-V3 | ARC-cf | AP100-88 | ARC-ff | 0 | 70 | 70 |
| LH06-082 | LB124 | 431-66 | ARC-rr | LH06-082 | ARC-ab | | | |
| | LB125 | LB124-5 | ARC-br | LB124-2 | ARC-br | | | |
| LH06-003 | LB128 | 833-8 | ARC-ff | LH06-003 | ARC-ir2 | | | |
| | LB140 | LB128-33 | ARC-fr2 | LB128-35 | ARC-fr2 | | | |
| | LB221 | LB207-43 | ARC-ff | LB140-41 | ARC-r2r2 | | | |
| | LB223 | LB140-53 | ARC-r2r2 | LB207-43 | ARC-ff | | | |
| | LB230 | LB221-1 | ARC-fr2 | LB221-2 | ARC-fr2 | | | |
| | LB232 | LB221-7 | ARC-fr2 | LB221-8 | ARC-fr2 | | | |
