# Supplementary material for: Genetic Background and Allorecognition Phenotype in Hydractinia symbiolongicarpus
Source: G3 (Bethesda). 2011 Nov 1;1(6):499–504. doi: 10.1534/g3.111.001149 (PMC3276163; doi:10.1534/g3.111.001149)
Supplement: Supporting Information [file supp_1.6.499_TableS3.pdf]

Table S3 Raw data[1]

Colony		Marker Genotype[3]												Phenotype against tester[4]			
		m194	m18	m28	m174	m29	f194	f174	alr2 exon 9	194c1	194c28	174il	217-11	833-8	AP100-88	4117-2	MP104-34
AP110,111	1	fd	fd	fd	fd												
AP110,111	2	fd	fd	fd	fd												
AP110,111	3	ff	ff	ff	ff									Fuse			
AP110,111	4	fd	fd	fd	fd												
AP110,111	5	ff	ff	ff	ff									Fuse			
AP110,111	6	ff	ff	ff	ff									Fuse			
AP110,111	7	fd	fd	fd	fd												
AP110,111	8	ff	ff	ff	ff									Fuse			
AP110,111	9	fd	fd	fd	fd												
AP110,111	10	fd	fd	fd	fd												
AP110,111	11	fd	fd	fd	fd												
AP110,111	12	ff	ff	ff	ff									Fuse			
AP110,111	13	ff	ff	ff	ff									Fuse			
AP110,111	14	ff	ff	ff	ff									Fuse			
AP110,111	15	ff	ff	ff	ff									Fuse			
AP110,111	16	fd	fd	fd	fd												
AP110,111	17	ff	ff	ff	ff									Fuse			
AP110,111	18	ff	fd	fd	fd												
AP110,111	19	fd	fd	fd	fd												
AP110,111	20	ff	ff	ff	ff									Fuse			
AP110,111	21	ff	ff	ff	ff									Fuse			
AP110,111	22	fd	fd	fd	fd												
AP110,111	23	ff	ff	ff	ff									Fuse			
AP110,111	24	ff	ff	ff	ff									Fuse			
AP110,111	25	fd	fd	fd	fd												
AP110,111	26	ff	ff	ff	ff									Fuse			
AP110,111	27	fd	fd	fd	fd												
AP110,111	28	ff	ff	ff	ff									Fuse			
AP110,111	29	fd	fd	ff	ff												
AP110,111	30	fd	fd	fd	fd												
AP110,111	31	ff	ff	ff	ff									Fuse			
AP110,111	32	ff	ff	ff	ff									Fuse			
AP110,111	33	ff	ff	ff	ff									Fuse			
AP110,111	34	ff	ff	ff	ff									Fuse			
AP110,111	35	fd	fd	fd	fd												

AP110,111	36	fd	fd	fd	fd	
AP110,111	37	fd	fd	fd	fd	
AP110,111	38	fd	fd	fd	fd	
AP110,111	39	fd	fd	fd	fd	
AP110,111	40	ff	ff	ff	ff	Fuse
AP110,111	41	fd	fd	fd	fd	
AP110,111	42	fd	fd	fd	fd	
AP110,111	43	fd	fd	fd	fd	
AP110,111	44	ff	ff	ff	ff	Fuse
AP110,111	45	fd	fd	fd	fd	
AP110,111	46	ff	ff	ff	ff	
AP110,111	47	ff	ff	ff	ff	
AP110,111	48	fd	fd	fd	fd	
AP110,111	49	ff	ff	ff	ff	
AP110,111	50	ff	ff	ff	ff	
AP110,111	51	fd	fd	fd	fd	
AP110,111	52	ff	ff	ff	ff	
AP110,111	53	fd	fd	fd	fd	
AP110,111	54		fd	fd	fd	
AP110,111	55	ff	ff	ff	ff	
AP110,111	56	ff	ff	ff	ff	
AP110,111	57	ff	ff	ff	ff	
AP110,111	58	ff	ff	ff	ff	
AP110,111	59	ff	ff	ff	ff	
AP110,111	60	fd	fd	fd	fd	
AP110,111	61		ff	ff	ff	
AP110,111	62	ff	ff	ff	ff	
AP110,111	63		fd	fd	fd	
AP110,111	64	fd	fd	fd	fd	
AP110,111	65	ff	ff	ff	ff	
AP110,111	66	fd	fd	fd	fd	
AP110,111	67	fd	fd	fd	fd	
AP110,111	68	ff	ff	ff	ff	
AP110,111	69	fd	fd	fd	fd	
AP110,111	70	ff	ff	ff	ff	
AP110,111	71	fd	fd	fd	fd	
AP110,111	72		fd	fd	fd	
AP110,111	73	fd	fd	fd	fd	
AP110,111	74	fd	fd	ff	ff	
AP110,111	75	fd	fd	fd	fd	
AP110,111	76	ff	ff	ff	ff	

AP110,111	77	fd	fd	fd	fd
AP110,111	78	fd	fd	fd	fd
AP110,111	79	fd	fd	fd	fd
AP110,111	80	fd	fd	fd	fd
AP110,111	81	fd	ff	ff	ff
AP110,111	82	fd	fd	fd	fd
AP110,111	83	ff	ff	ff	ff
AP110,111	84	ff	ff	ff	ff
AP110,111	85		ff	ff	ff
AP110,111	86		ff	ff	ff
AP110,111	87	fd	fd	fd	fd
AP110,111	88	fd	fd	fd	fd
AP110,111	89		fd	fd	fd
AP110,111	90	ff	ff	ff	ff
AP110,111	91	ff	ff	ff	ff
AP110,111	92	ff	ff	ff	ff
AP110,111	93	ff	ff	ff	ff
AP110,111	94	fd	fd	fd	fd
AP110,111	95	fd	fd	fd	fd
AP110,111	96	ff	ff	ff	ff
AP110,111	97		ff	ff	ff
AP110,111	98	fd	fd	fd	fd
AP110,111	99	ff	ff	ff	ff
AP110,111	100	ff	ff	ff	ff
AP110,111	101	ff	ff	fd	fd
AP110,111	102	ff	ff	ff	ff
AP110,111	103	fd	fd	fd	fd
AP110,111	104	ff	ff	ff	ff
AP110,111	105	fd	fd	fd	fd
AP110,111	106	ff	ff	ff	ff
AP110,111	107	ff	ff	ff	ff
AP110,111	108	ff	ff	ff	ff
AP110,111	109	ff	ff	ff	ff
AP110,111	110		fd	fd	fd
AP110,111	111	ff	ff	ff	ff
AP110,111	112	fd	fd	fd	fd
AP110,111	113	ff	ff	ff	ff
AP110,111	114	ff	ff	ff	ff
AP110,111	115	ff	ff	ff	ff
AP110,111	116	fd	fd	fd	fd
AP110,111	117		fd	fd	fd

AP110,111	118	ff	ff	ff	ff
AP110,111	119	fd	fd	fd	fd
AP110,111	120	ff	ff	ff	ff
AP110,111	121	fd	fd	fd	fd
AP110,111	122	ff	ff	ff	ff
AP110,111	123	ff	ff	ff	ff
AP110,111	124	fd	fd	fd	fd
AP110,111	125	fd	fd	fd	fd
AP110,111	126	fd	fd	fd	fd
AP110,111	127	ff	ff	ff	ff
AP110,111	128	ff	ff	ff	ff
AP110,111	129	fd		fd	fd
AP110,111	130	ff	ff	ff	ff
AP110,111	131	fd	fd	fd	fd
AP110,111	132	fd	fd	fd	fd
AP110,111	133	ff	ff	ff	ff
AP110,111	134	fd	fd	fd	fd
AP110,111	135	ff	ff	ff	ff
AP110,111	136		fd	fd	ff
AP110,111	137	ff	ff	ff	ff
AP110,111	138	fd	fd	fd	fd
AP110,111	139	ff	ff	ff	ff
AP110,111	140	ff	ff	ff	ff
AP110,111	141	ff	ff	ff	ff
AP110,111	142		fd	fd	fd
AP110,111	143		fd	fd	fd
AP110,111	144	fd	fd	fd	fd
AP110,111	145	fd	fd	fd	fd
AP110,111	146	fd	fd	fd	fd
AP110,111	147	fd		fd	fd
AP110,111	148	fd	fd	fd	fd
AP110,111	149		ff	ff	ff
AP110,111	150	fd	fd	fd	fd
AP110,111	151	ff	ff	ff	ff
AP110,111	152		fd	fd	fd
AP110,111	153	fd	fd	fd	fd
AP110,111	154		ff	ff	ff
AP110,111	155		ff	ff	ff
AP110,111	156		fd	fd	fd
AP110,111	157	fd	fd	fd	fd
AP110,111	158		ff	fd	ff

AP110,111	159		fd	fd	fd
AP110,111	160		ff	ff	ff
AP110,111	161	fd	fd	fd	fd
AP110,111	162	fd	ff	fd	fd
AP110,111	163	ff	ff	ff	ff
AP110,111	164		ff	ff	ff
AP110,111	165		fd	fd	fd
AP110,111	166	fd	fd	fd	
AP110,111	167	ff	ff	ff	ff
AP110,111	168	fd	fd	fd	fd
AP110,111	169	ff	ff	fd	fd
AP110,111	170	ff	ff	ff	ff
AP110,111	171	fd		fd	fd
AP110,111	172	ff	ff	ff	ff
AP110,111	173	ff	ff	ff	ff
AP110,111	174		fd	fd	fd
AP110,111	175		ff	ff	ff
AP110,111	176		ff	ff	ff
AP110,111	177		ff	ff	ff
AP110,111	178	ff	ff	ff	fd
AP110,111	179	ff	fd	fd	fd
AP110,111	180	ff	ff	ff	ff
AP110,111	181	fd	fd	fd	fd
AP110,111	182		ff	ff	ff
AP110,111	183	fd	ff	ff	ff
AP110,111	184	fd	fd	fd	fd
AP110,111	185	fd	fd	fd	fd
AP110,111	186	fd	fd	fd	fd
AP110,111	187	ff	ff	ff	ff
AP110,111	188	fd	fd	fd	fd
AP110,111	189	ff	ff	ff	ff
AP110,111	190		fd	fd	fd
AP110,111	191		fd	fd	fd
AP110,111	192		ff	ff	ff
AP110,111	193	ff	fd	fd	fd
AP110,111	194		fd	fd	fd
AP110,111	195		ff	ff	ff
AP110,111	196		fd	fd	fd
AP110,111	197	fd	fd	fd	fd
AP110,111	198	ff	ff	ff	ff
AP110,111	199		fd	fd	fd

AP110,111	200	ff	ff	ff	ff
AP110,111	201	ff	ff	ff	ff
AP110,111	202		fd	fd	fd
AP110,111	203	ff	ff		ff
AP110,111	204	ff	ff	ff	ff
AP110,111	205	fd	fd	fd	fd
AP110,111	206		fd	fd	fd
AP110,111	207	fd	fd	fd	
AP110,111	208		ff	ff	ff
AP110,111	209	fd	fd	fd	fd
AP110,111	210		fd	fd	fd
AP110,111	211		ff	ff	ff
AP110,111	212		fd	fd	fd
AP110,111	213	fd	fd	fd	fd
AP110,111	214	ff	ff		ff
AP110,111	215	fd	fd	fd	fd
AP110,111	216	fd	fd	fd	fd
AP110,111	217		ff	ff	ff
AP110,111	218	fd	fd	fd	fd
AP110,111	219	ff	ff	ff	ff
AP110,111	220	ff	ff	ff	ff
AP110,111	221	fd	fd	fd	
AP110,111	222	ff	fd	fd	fd
AP110,111	223		fd	fd	fd
AP110,111	224		fd	fd	fd
AP110,111	225		fd	fd	fd
AP110,111	226	ff	ff	ff	fd
AP110,111	227		fd	fd	fd
AP110,111	228	ff	ff	ff	
AP110,111	229	ff	ff	ff	ff
AP110,111	230	ff	ff	ff	ff
AP110,111	231	fd	fd	fd	ff
AP110,111	232		fd	fd	fd
AP110,111	233	ff	ff	ff	ff
AP110,111	234		fd	ff	ff
AP110,111	235	fd	fd	fd	fd
AP110,111	236	fd	fd	fd	fd
AP110,111	237	fd		ff	ff
AP110,111	238	ff	ff	ff	ff
AP110,111	239		fd	ff	ff
AP110,111	240	ff	ff	ff	ff

AP110,111	241	ff	fd	fd	ff
AP110,111	242	ff	ff	ff	ff
AP110,111	243	ff	ff	ff	ff
AP110,111	244	ff	ff	ff	ff
AP110,111	245	fd	fd	fd	fd
AP110,111	246	fd	fd	fd	fd
AP110,111	247		fd	fd	fd
AP110,111	248		fd	ff	fd
AP110,111	249	ff	ff	ff	ff
AP110,111	250	ff	fd	ff	
AP110,111	251	ff	ff	ff	ff
AP110,111	252		fd	fd	fd
AP110,111	253	fd	fd	fd	
AP110,111	254	fd	fd		fd
AP110,111	255		ff	ff	ff
AP110,111	256		fd	fd	fd
AP110,111	257		fd	fd	fd
AP110,111	258	ff	ff	ff	ff
AP110,111	259		ff	ff	ff
AP110,111	260	ff	ff	ff	ff
AP110,111	261	ff	ff	ff	ff
AP110,111	262		fd	fd	fd
AP110,111	263	fd		fd	fd
AP110,111	264	ff	fd	fd	
AP110,111	265	fd	fd	fd	fd
AP110,111	266		fd	fd	fd
AP110,111	267	fd	fd	fd	fd
AP110,111	268		fd	fd	fd
AP110,111	269	fd	fd	fd	fd
AP110,111	270	ff	fd	fd	fd
AP110,111	271	fd	fd	fd	fd
AP110,111	272	ff	ff	ff	ff
AP110,111	273	ff	ff	ff	ff
AP110,111	274	fd	fd	fd	fd
AP110,111	275		ff	ff	ff
AP110,111	276	ff	ff	ff	ff
AP110,111	277	fd	ff	ff	
AP110,111	278	fd	fd	fd	fd
AP110,111	279		ff	ff	fd
AP110,111	280		ff	ff	ff
AP110,111	281	ff	ff	ff	

AP110,111	282	ff	ff	ff	ff
AP110,111	283	ff	ff	ff	ff
AP110,111	284	fd	fd	fd	fd
AP110,111	285	fd	fd	fd	fd
AP110,111	286	fd	fd	fd	fd
AP110,111	287	fd	fd	fd	fd
AP110,111	288	ff	ff	ff	ff
AP110,111	289	fd	fd	fd	fd
AP110,111	290	ff	ff	ff	ff
AP110,111	291		fd	fd	fd
AP110,111	292	ff	ff	ff	ff
AP110,111	293	fd	fd	fd	fd
AP110,111	294	ff	ff	ff	ff
AP110,111	295	fd	fd	fd	fd
AP110,111	296	ff	ff	ff	ff
AP110,111	297	ff	ff	ff	ff
AP110,111	298	ff	ff	ff	ff
AP110,111	299		ff	ff	ff
AP110,111	300	fd	fd	fd	fd
AP110,111	301	fd	fd	fd	fd
AP110,111	302	fd	fd	fd	fd
AP110,111	303		ff	ff	ff
AP110,111	304	fd	fd	fd	fd
AP110,111	305	fd	fd	fd	fd
AP110,111	306	ff	ff	ff	ff
AP110,111	307	fd	fd	fd	fd
AP110,111	308	fd	fd	fd	fd
AP110,111	309	ff	ff	ff	ff
AP110,111	310	ff	ff	ff	ff
AP110,111	311	ff	ff	ff	ff
AP110,111	312	ff	ff	ff	ff
AP110,111	313	ff	ff	ff	ff
AP110,111	314	fd	fd	fd	fd
AP110,111	315	ff	ff	ff	ff
AP110,111	316	ff	ff	ff	ff
AP110,111	317	ff	ff	ff	ff
AP110,111	318	ff	ff	ff	ff
AP110,111	319	ff	ff	ff	ff
AP110,111	320	ff	ff	ff	ff
AP110,111	321		fd	fd	fd
AP110,111	322	fd	fd	fd	fd

AP110,111	323	fd	fd	fd	fd
AP110,111	324	ff	ff	ff	ff
AP110,111	325	ff	ff	ff	ff
AP110,111	326	fd	fd	fd	fd
AP110,111	327	ff	ff	ff	ff
AP110,111	328	ff	ff	ff	ff
AP110,111	329	fd	fd	fd	fd
AP110,111	330	ff	fd	fd	fd
AP110,111	331	fd	fd	fd	fd
AP110,111	332	ff	ff	ff	ff
AP110,111	333	ff	fd	fd	fd
AP110,111	334		fd	fd	fd
AP110,111	335	ff	ff	ff	ff
AP110,111	336	ff	ff	ff	ff
AP110,111	337	ff	ff	ff	ff
AP110,111	338	ff	ff	ff	ff
AP110,111	339	ff	ff	ff	ff
AP110,111	340	ff	ff	ff	ff
AP110,111	341	fd	fd	fd	fd
AP110,111	342		fd	fd	fd
AP110,111	343	ff	ff	ff	ff
AP110,111	344	ff	ff	ff	ff
AP110,111	345	ff	ff	ff	ff
AP110,111	346	ff	ff	ff	ff
AP110,111	347		fd	fd	fd
AP110,111	348	ff	ff	ff	ff
AP110,111	349	fd	fd	fd	fd
AP110,111	350	fd	fd	fd	fd
AP110,111	351	ff	ff	ff	
AP110,111	352	ff	ff	ff	ff
AP110,111	353	fd	fd	fd	fd
AP110,111	354	ff	ff	ff	ff
AP110,111	355	ff		ff	ff
AP110,111	356	ff	ff	ff	ff
AP110,111	357	ff	ff	ff	ff
AP110,111	358	fd	fd	fd	fd
AP110,111	359	fd	fd	fd	fd
AP110,111	360	fd	fd	fd	fd
AP110,111	361	fd	fd	fd	fd
AP110,111	362	ff	ff	ff	ff
AP110,111	363	fd	fd	fd	fd

AP110,111	364	ff	ff	ff	ff
AP110,111	365	fd	fd	fd	fd
AP110,111	366	fd	ff	ff	ff
AP110,111	367	ff	ff	ff	ff
AP110,111	368		ff	ff	ff
AP110,111	369	fd	fd	fd	fd
AP110,111	370	ff	ff	ff	ff
AP110,111	371		fd	fd	fd
AP110,111	372	fd	fd	fd	fd
AP110,111	373		fd	fd	fd
AP110,111	374		fd	fd	fd
AP110,111	375	fd	fd	fd	fd
AP110,111	376	fd	fd	fd	fd
AP110,111	377	fd	fd	fd	fd
AP110,111	378	ff	ff	ff	ff
AP110,111	379	fd	fd	fd	fd
AP110,111	380	ff	ff	ff	ff
AP110,111	381	ff	ff	ff	ff
AP110,111	382	ff	ff	ff	ff
AP110,111	383	ff	ff	ff	ff
AP110,111	384	ff	ff	ff	ff
AP110,111	385	ff	ff	ff	ff
AP110,111	386	fd	fd	fd	fd
AP110,111	387	fd	fd	fd	fd
AP110,111	388	fd	fd	fd	fd
AP110,111	389	ff	ff	ff	ff
AP110,111	390		ff	ff	ff
AP110,111	391	ff	ff	ff	ff
AP110,111	392	fd	fd	fd	fd
AP110,111	393	fd	fd	fd	fd
AP110,111	394		fd	fd	fd
AP110,111	395	ff	ff	ff	ff
AP110,111	396	fd	fd	fd	
AP110,111	397	ff	ff	ff	ff
AP110,111	398	fd	fd	fd	fd
AP110,111	399	fd	fd	fd	
AP110,111	400	fd	fd	fd	fd
AP110,111	401		ff	ff	ff
AP110,111	402	fd	fd	fd	fd
AP110,111	403	ff	ff	ff	ff
AP110,111	404	fd	ff	ff	ff

AP110,111	405	fd	fd	fd	fd
AP110,111	406		fd	fd	fd
AP110,111	407	fd	fd	fd	fd
AP110,111	408	fd	fd	fd	fd
AP110,111	409		fd	fd	fd
AP110,111	410	ff	ff	ff	ff
AP110,111	411	fd	fd	fd	fd
AP110,111	412	ff	ff	ff	ff
AP110,111	413	ff	ff	ff	ff
AP110,111	414	ff	ff	ff	ff
AP110,111	415	ff	ff	ff	ff
AP110,111	416	fd		fd	fd
AP110,111	417		ff	ff	ff
AP110,111	418	fd	ff	ff	ff
AP110,111	419	ff	ff	ff	ff
AP110,111	420	ff	ff	ff	ff
AP110,111	421	ff	fd	fd	fd
AP110,111	422		fd	fd	fd
AP110,111	423	fd	fd	fd	fd
AP110,111	424	ff	ff		ff
AP110,111	425	ff	ff	ff	ff
AP110,111	426	fd	fd	fd	fd
AP110,111	427	fd	fd	fd	fd
AP110,111	428		fd	fd	fd
AP110,111	429	fd	fd	fd	fd
AP110,111	430	ff	ff	ff	ff
AP110,111	431	fd	fd	fd	fd
AP110,111	432	fd	fd	fd	fd
AP110,111	433		fd	fd	fd
AP110,111	434	ff	ff	ff	ff
AP110,111	435	ff	ff	ff	ff
AP110,111	436	fd	fd	fd	fd
AP110,111	437	fd	fd	fd	fd
AP110,111	438	fd	fd	fd	fd
AP110,111	439	fd	fd	fd	fd
AP110,111	440	fd	fd	fd	fd
AP110,111	441	fd	fd	fd	fd
AP110,111	442	fd	fd	fd	fd
AP110,111	443	fd		ff	ff
AP110,111	444	ff	ff	ff	ff
AP110,111	445	fd	fd	fd	fd

AP110,111	446	fd	fd	fd	fd
AP110,111	447	fd	fd	fd	fd
AP110,111	448	fd	fd	fd	fd
AP110,111	449	ff	ff	ff	ff
AP110,111	450	ff	ff	ff	ff
AP110,111	451	fd	fd	fd	fd
AP110,111	452	ff	ff	ff	ff
AP110,111	453	ff	ff	ff	ff
AP110,111	454	fd	fd	fd	fd
AP110,111	455	fd	fd	fd	fd
AP110,111	456		fd	fd	fd
AP110,111	457	ff		ff	ff
AP110,111	458	fd	fd	fd	fd
AP110,111	459	ff		ff	ff
AP110,111	460	ff	ff	ff	ff
AP110,111	461	ff	ff	ff	ff
AP110,111	462	ff		ff	ff
AP110,111	463	ff	ff		fd
AP110,111	464		fd	fd	fd
AP110,111	465	fd	fd	fd	fd
AP110,111	466		fd	fd	fd
AP110,111	467	ff	ff	ff	ff
AP110,111	468	ff	ff	fd	fd
AP110,111	469	ff	ff	ff	ff
AP110,111	470	ff	ff	ff	ff
AP110,111	471	ff	ff	ff	ff
AP110,111	472	ff	ff	ff	ff
AP110,111	473	fd	fd	fd	fd
AP110,111	474		fd	fd	fd
AP110,111	475	ff	ff	ff	ff
AP110,111	476	ff	fd	fd	fd
AP110,111	477	ff	ff	ff	ff
AP110,111	478		fd	fd	fd
AP110,111	479	ff	ff	ff	ff
AP110,111	480		fd	fd	fd
AP110,111	481	ff	fd	fd	fd
AP110,111	482	fd	fd	fd	
AP110,111	483	ff	ff	ff	ff
AP110,111	484	ff	ff	ff	ff
AP110,111	485	ff	ff	ff	ff
AP110,111	486	fd	fd	fd	fd

AP110,111	487	ff	ff	ff	ff
AP110,111	488		fd	fd	fd
AP110,111	489	ff	fd	fd	fd
AP110,111	490	ff	ff	ff	ff
AP110,111	491	fd	fd	fd	fd
AP110,111	492	ff	ff	ff	ff
AP110,111	493	fd	fd	fd	fd
AP110,111	494	ff	ff	ff	ff
AP110,111	495	ff	ff	ff	ff
AP110,111	496	ff	ff	ff	ff
AP110,111	497	ff	ff	ff	ff
AP110,111	498	fd	fd	fd	fd
AP110,111	499	ff	ff	ff	ff
AP110,111	500	fd	fd	fd	fd
AP110,111	501	ff	ff	ff	ff
AP110,111	502		ff	ff	ff
AP110,111	503	ff	ff	ff	ff
AP110,111	504	fd	fd	fd	fd
AP110,111	505	fd	fd	ff	ff
AP110,111	506	fd	fd	fd	fd
AP110,111	507	ff	ff	ff	ff
AP110,111	508	ff	ff	ff	ff
AP110,111	509	fd	fd	fd	fd
AP110,111	510	fd	fd	fd	fd
AP110,111	511	fd	fd	fd	fd
AP110,111	512	ff	ff	ff	ff
AP110,111	513	fd	fd	fd	fd
AP110,111	514	fd	fd	fd	fd
AP110,111	515	fd	fd	fd	fd
AP110,111	516	ff	ff	ff	ff
AP110,111	517	ff	fd	ff	ff
AP110,111	518		fd	fd	fd
AP110,111	519	ff	ff	ff	ff
AP110,111	520	fd	fd	fd	fd
AP110,111	521	ff	ff	ff	ff
AP110,111	522	ff	ff	ff	ff
AP110,111	523	fd	fd	fd	fd
AP110,111	524	ff	ff	ff	ff
AP110,111	525	ff	ff	ff	ff
AP110,111	526	ff	ff	ff	ff
AP110,111	527	ff	ff	ff	ff

AP110,111	528	fd	fd	fd	fd
AP110,111	529	fd	fd	fd	fd
AP110,111	530	ff	ff	ff	ff
AP110,111	531		ff	ff	ff
AP110,111	532	ff	ff	ff	ff
AP110,111	533	fd	fd	fd	fd
AP110,111	534	fd	fd	fd	fd
AP110,111	535	ff	ff	ff	ff
AP110,111	536	fd	fd	fd	fd
AP110,111	537		ff	ff	ff
AP110,111	538	fd	fd	fd	fd
AP110,111	539	ff	ff	ff	ff
AP110,111	540	ff	fd	fd	ff
AP110,111	541	ff	ff	ff	ff
AP110,111	542	ff	ff	ff	ff
AP110,111	543	ff	ff	ff	ff
AP110,111	544	fd	fd	fd	fd
AP110,111	545	fd	fd	fd	fd
AP110,111	546	ff	ff	ff	ff
AP110,111	547	ff	ff	ff	ff
AP110,111	548	fd		fd	fd
AP110,111	549	fd	fd	fd	fd
AP110,111	550	fd	fd	fd	fd
AP110,111	551	ff	ff	ff	ff
AP110,111	552		fd	fd	fd
AP110,111	553	fd	fd	fd	fd
AP110,111	554	ff	ff	ff	ff
AP110,111	555	ff	ff	ff	ff
AP110,111	556	fd	fd	fd	fd
AP110,111	557	ff	ff	ff	ff
AP110,111	558	fd	fd	fd	fd
AP110,111	559	fd	fd	fd	fd
AP110,111	560	ff	ff	ff	ff
AP110,111	561		fd	fd	fd
AP110,111	562	fd	fd	fd	fd
AP110,111	563	fd	fd	fd	fd
AP110,111	564	ff	ff	ff	ff
AP110,111	565	ff	ff	ff	ff
AP110,111	566	fd	ff	ff	ff
AP110,111	567	ff	ff	ff	ff
AP110,111	568	ff	ff	ff	ff

AP110,111	569	fd	fd	fd	fd	
AP110,111	570	fd	fd	fd	fd	
AP110,111	571		ff	ff	ff	
AP110,111	572	ff	ff	ff	ff	
AP110,111	573		ff	ff	ff	
AP110,111	574	ff	fd	fd	fd	
AP110,111	575	ff	ff	ff	ff	
AP110,111	576	ff	fd	ff	ff	
AP110,111	577		fd	fd	fd	
AP110,111	578	fd	fd	fd	fd	
AP110,111	579		ff	ff	ff	
AP110,111	580		fd	fd	fd	
AP110,111	581		ff	ff	ff	
AP110,111	582		fd	fd	fd	
AP110,111	583		fd	fd	fd	
AP110,111	584		ff	ff	ff	
AP110,111	585		fd	fd	fd	
AP110,111	586		ff	ff	ff	
AP110,111	587	fd	fd	fd	fd	
AP110,111	588	fd	fd	fd	fd	
AP110,111	589		fd	fd	fd	
AP110,111	590	fd	fd	fd	fd	
AP110,111	591		fd	fd	fd	
AP110,111	592	ff	ff	ff	ff	
AP110,111	593	ff	ff	ff	ff	
AP110,111	594	ff	ff	ff	ff	
AP110,111	595	ff	ff	ff	ff	
AP110,111	596	fd	fd	fd	fd	
AP110,111	597		fd	fd	fd	
AP110,111	598		ff	ff	ff	
AP110,111	599		ff	ff	ff	
AP110,111	600	fd	fd	fd	fd	
AP110,111	601	ff	ff	ff	ff	
AP110,111	602	ff	ff	ff	ff	
AP105	1	fc	fc	fc	fc	
AP105	2	fc	fc	fc	fc	
AP105	3	ff	fc	fc	fc	
AP105	4	ff	ff	ff	ff	Fuse
AP105	5	ff	ff	ff	ff	Fuse
AP105	6	fc	fc	fc	fc	
AP105	7	fc	fc	fc	fc	

AP105	8	fc	ff	ff	ff	ff	
AP105	9	ff	ff	ff	ff	ff	Fuse
AP105	10	ff	ff	ff	ff	ff	Fuse
AP105	11	fc	ff	ff	ff	ff	
AP105	12	fc	fc	fc	fc	ff	
AP105	13	ff	ff	ff	ff	ff	Fuse
AP105	14	ff	ff	ff	ff	ff	Fuse
AP105	15	ff	ff	ff	ff	ff	
AP105	16	fc	ff	ff	ff	ff	
AP105	17	ff	ff	ff	ff	ff	Fuse
AP105	18	ff	ff	ff	ff	ff	Fuse
AP105	19	ff	ff	ff	ff	ff	Fuse
AP105	20	ff	ff	ff	ff	ff	
AP105	21	ff	ff	ff	ff	ff	
AP105	22	ff	ff	ff	ff	ff	
AP105	23	fc	ff	ff	ff	ff	
AP105	24	fc	ff	ff	ff	ff	
AP105	25	ff	ff	ff	ff	ff	
AP105	26	ff	ff	ff	ff	ff	
AP105	27	ff	ff	ff	ff	ff	
AP105	28	ff	ff	ff	ff	ff	
AP105	29	ff	ff	ff	ff	ff	
AP105	30	ff	ff	ff	ff	ff	
AP105	31	ff	ff	ff	ff	ff	
AP105	32	ff	ff	ff	ff	ff	
AP105	33		ff	ff	ff	ff	
AP105	34		ff	ff	ff	ff	
AP105	35	ff	ff	ff	ff	ff	
AP105	36		ff	ff	ff	ff	
AP105	37		ff	ff	ff	ff	
AP105	38		ff	ff	ff	ff	
AP105	39	ff	ff	ff	ff	ff	
AP105	40	ff	ff	ff	ff	ff	
AP105	41	ff	ff	ff	ff	ff	
AP105	42	fc	fc	ff	ff	ff	
AP105	43	fc	ff	ff	ff	ff	
AP105	44	ff	ff	ff	ff	ff	
AP105	45		ff	ff	ff	ff	
AP105	46	ff	ff	ff	ff	ff	
AP105	47	ff	ff	ff	ff	ff	
AP105	48	ff	ff	ff	ff	ff	

AP105	49	ff	ff	ff	ff	ff
AP105	50	ff	ff	ff	ff	ff
AP105	51	ff	ff	ff	ff	ff
AP105	52	ff	ff	ff	ff	ff
AP105	53	ff	ff	ff	ff	ff
AP105	54	ff	ff	ff	ff	ff
AP105	55	ff	ff	ff	ff	ff
AP105	56	ff	ff	ff	ff	ff
AP105	57		ff	ff	ff	ff
AP105	58	ff	ff	ff	ff	ff
AP105	59	ff	ff	ff	ff	ff
AP105	60	ff	ff	ff	ff	ff
AP105	61	ff	ff	ff	ff	ff
AP105	62	ff	ff	ff	ff	ff
AP105	63		ff	ff	ff	ff
AP105	64	fc	fc	ff	ff	ff
AP105	65	ff	ff	ff	ff	ff
AP105	66	ff	ff	ff	ff	ff
AP105	67	ff	ff	ff	ff	ff
AP105	68	ff	ff	ff	ff	ff
AP105	69		ff	ff	ff	ff
AP105	70		ff	ff	ff	ff
AP105	71	ff	ff	ff	ff	ff
AP105	72	ff	ff	ff	ff	ff
AP105	73	ff	ff	ff	ff	ff
AP105	74	ff	ff	ff	ff	ff
AP105	75	ff	ff	ff	ff	ff
AP105	76	ff	ff	ff	ff	ff
AP105	77	ff	ff	ff	ff	ff
AP105	78	ff	ff	ff	ff	ff
AP105	79	ff	ff	ff	ff	ff
AP105	80	fc	fc	fc	fc	ff
AP105	81		ff	ff	ff	ff
AP105	82	ff	fc	ff	ff	ff
AP105	83	ff	ff	ff	ff	ff
AP105	84	ff	ff	ff	ff	ff
AP105	85	fc	fc	fc	fc	ff
AP105	86		ff	ff	ff	ff
AP105	87	ff	ff	ff	ff	ff
AP105	88	ff	ff	ff	ff	ff
AP105	89	fc	ff	ff	ff	ff

AP105	90		ff	ff	ff	ff
AP105	91		fc	fc	fc	ff
AP105	92	fc	ff	ff	ff	ff
AP105	93	ff	ff	ff	ff	ff
AP105	94		ff	ff	ff	ff
AP105	95	fc	fc	fc	fc	ff
AP105	96		fc	ff	ff	ff
AP105	97		ff	ff	ff	ff
AP105	98	fc	fc	fc	fc	ff
AP105	99		ff	ff	fc	ff
AP105	100		ff	ff	ff	ff
AP105	101		fc	fc	fc	ff
AP105	102	ff	ff	ff	ff	ff
AP105	103		fc	ff	ff	ff
AP105	104		ff	ff	ff	ff
AP105	105	ff	ff	ff	fc	ff
AP105	106	ff	ff	ff	ff	ff
AP105	107		ff	ff	ff	ff
AP105	108	ff	ff	ff	ff	ff
AP105	109		ff	ff	ff	ff
AP105	110	fc	ff	ff	ff	ff
AP105	111	ff	ff		ff	ff
AP105	112		ff	ff	ff	ff
AP105	113	fc	ff	ff	ff	ff
AP105	114	ff	fc	ff	fc	ff
AP105	115	ff	ff	ff	ff	ff
AP105	116	fc	ff	ff	ff	ff
AP105	117	ff	ff	ff	ff	ff
AP105	118	ff	ff	ff	ff	ff
AP105	119	fc	ff	ff	ff	ff
AP105	120	ff	ff	ff	ff	ff
AP105	121	ff	ff	ff	ff	ff
AP105	122	ff	ff	ff	ff	ff
AP105	123	fc	ff	ff	ff	ff
AP105	124	ff		ff	ff	ff
AP105	125		fc	ff	ff	ff
AP105	126	ff	fc	ff	ff	ff
AP105	127		fc	fc	fc	ff
AP105	128		ff	ff	ff	ff
AP105	129		ff	ff	ff	ff
AP105	130	ff	ff	ff		ff

AP105	131	ff	ff	fc	ff	ff
AP105	132		ff	ff	fc	ff
AP105	133	ff	ff	ff	ff	ff
AP105	134		ff	ff	ff	ff
AP105	135	fc	fc	fc	fc	ff
AP105	136	ff	ff	ff	ff	ff
AP105	137	ff	ff	ff	ff	ff
AP105	138		ff	ff	ff	ff
AP105	139	ff	ff	ff	ff	ff
AP105	140	ff	ff	ff	ff	ff
AP105	141		ff	ff	ff	ff
AP105	142		ff	ff	ff	ff
AP105	143	ff	ff	ff	ff	ff
AP105	144	ff	ff	ff	ff	ff
AP105	145	ff	ff	ff	ff	ff
AP105	146	ff	ff	ff	ff	ff
AP105	147		ff	ff	ff	ff
AP105	148	ff	ff	ff	ff	ff
AP105	149	fc	ff	ff	ff	ff
AP105	150	ff	ff	ff	ff	ff
AP105	151	fc	ff	ff	ff	ff
AP105	152	ff	ff	ff	ff	ff
AP105	153	fc	ff	ff	ff	ff
AP105	154		ff	ff	ff	ff
AP105	155		ff	ff	ff	ff
AP105	156		ff	ff	ff	ff
AP105	157	fc	ff	ff	ff	ff
AP105	158	ff	ff	ff	ff	ff
AP105	159		ff	ff	ff	ff
AP105	160	ff	ff	ff	ff	ff
AP105	161	fc	fc	fc	fc	fc
AP105	162	fc	fc	fc	fc	fc
AP105	163	fc	fc	fc	fc	fc
AP105	164	fc	fc	fc		fc
AP105	165	fc	fc	fc	fc	fc
AP105	166	fc	fc	fc	fc	fc
AP105	167	fc	fc	fc	fc	fc
AP105	168	fc	fc	fc	fc	fc
AP105	169	ff	fc	fc	fc	fc
AP105	170	fc	fc	fc	fc	fc
AP105	171	ff	ff	ff	ff	fc

AP105	172	fc	fc	fc	fc	fc
AP105	173	fc	fc	fc	fc	fc
AP105	174	fc	fc	fc	fc	fc
AP105	175	fc	fc	fc	fc	fc
AP105	176	ff	fc	fc	fc	fc
AP105	177	fc	fc	fc	fc	fc
AP105	178	fc	fc	fc	fc	fc
AP105	179	fc	fc	fc	fc	fc
AP105	180	fc	fc	fc	fc	fc
AP105	181	fc	fc	fc	fc	fc
AP105	182	ff	fc	fc	fc	fc
AP105	183	fc	fc	fc	fc	fc
AP105	184	fc	fc	fc	fc	fc
AP105	185	fc	fc	fc	fc	fc
AP105	186	fc	fc	fc	fc	fc
AP105	187	fc	fc	fc	fc	fc
AP105	188	fc	fc	fc	fc	fc
AP105	189	ff	ff	ff	ff	fc
AP105	190	fc	fc	fc	fc	fc
AP105	191	fc	fc	fc	fc	fc
AP105	192	fc	fc	fc	fc	fc
AP105	193		fc	fc	fc	fc
AP105	194	fc	fc	fc	fc	fc
AP105	195	fc	fc	fc	fc	fc
AP105	196	ff	fc	fc	fc	fc
AP105	197	fc	fc	fc	fc	fc
AP105	198	fc	fc	fc	fc	fc
AP105	199	ff	fc	fc	fc	fc
AP105	200		fc	fc	fc	fc
AP105	201	fc	fc	fc	fc	fc
AP105	202	fc	fc	fc	fc	fc
AP105	203	ff	ff	fc	fc	fc
AP105	204	fc	fc	fc	fc	fc
AP105	205	ff	ff	fc	fc	fc
AP105	206	fc	fc	fc	fc	fc
AP105	207	fc	fc	fc	fc	fc
AP105	208	fc	fc	fc	fc	fc
AP105	209	fc	fc	fc	fc	fc
AP105	210	fc	fc	fc	fc	fc
AP105	211	fc	fc	fc	fc	fc
AP105	212		fc	fc	fc	fc

AP105	213	ff	fc	fc	fc	fc
AP105	214	fc	fc	fc	fc	fc
AP105	215	fc	fc	fc	fc	fc
AP105	216	ff	ff	fc	fc	fc
AP105	217	fc	fc	fc	fc	fc
AP105	218	ff	fc	fc	fc	fc
AP105	219	ff	ff	fc	fc	fc
AP105	220		fc	fc	fc	fc
AP105	221	fc	fc	fc	fc	fc
AP105	222	fc	fc	fc	fc	fc
AP105	223	fc	fc	fc	fc	fc
AP105	224	fc	fc	fc	fc	fc
AP105	225	ff	ff	fc	fc	fc
AP105	226		fc	fc	fc	fc
AP105	227	fc	fc	fc	fc	fc
AP105	228	fc	fc	fc	fc	fc
AP105	229	fc	fc	fc	fc	fc
AP105	230	fc	fc	fc	fc	fc
AP105	231	ff	fc	fc	fc	fc
AP105	232	fc	fc	fc		fc
AP105	233	fc	fc	fc	fc	fc
AP105	234	ff	fc	fc	fc	fc
AP105	235	fc	fc	fc	fc	fc
AP105	236		ff	ff	ff	fc
AP105	237	fc	fc	fc	fc	fc
AP105	238	fc	fc	fc	fc	fc
AP105	239		fc	fc	fc	fc
AP105	240	ff	ff	ff	ff	fc
AP105	241	fc	fc	fc	fc	fc
AP105	242	ff	fc	fc	fc	fc
AP105	243	ff	fc	fc	fc	fc
AP105	244		fc	fc	fc	fc
AP105	245		fc	fc	fc	fc
AP105	246		ff	ff	ff	fc
AP105	247	fc	fc	fc	ff	fc
AP105	248	fc	fc	fc	fc	fc
AP105	249	fc	fc	fc	fc	fc
AP105	250	ff	fc	fc	fc	fc
AP105	251	ff	fc	fc	fc	fc
AP105	252		fc	fc	fc	fc
AP105	253		fc	fc	fc	fc

AP105	254	ff	ff	fc	fc	fc			
AP105	255	fc	fc	fc	fc	fc			
AP105	256	fc	ff	ff	ff	fc			
AP105	257	ff	fc	fc	fc	fc			
AP105	258		ff	ff	ff	fc			
AP105	259	ff	ff	fc	fc	fc			
AP105	260	ff	ff	ff	ff	fc			
AP105	261	fc	fc	fc	fc	fc			
AP105	262	ff	fc	fc	fc	fc			
AP105	263	ff	ff	ff	ff	fc			
AP105	264		ff	ff	ff	fc			
AP105	265		ff	ff	ff	fc			
AP105	266	ff	fc	fc	fc	fc			
AP105	267	fc	fc	fc	fc	fc			
AP105	268		fc	fc	fc	fc			
AP105	269		fc	fc	fc	fc			
AP105	270	fc	fc	fc	fc	fc			
AP105	271		fc	fc	fc	fc			
AP105	272		fc	fc	fc	fc			
AP105	273	ff	fc	fc	fc	fc			
AP105	274	ff	fc	fc	fc	fc			
AP105	275		fc	fc	fc	fc			
AP105	276	ff	fc	fc	fc	fc			
AP105	277	fc	fc	fc	fc	fc			
AP105	278	fc	fc	fc	fc	fc			
AP105	279	fc	fc	fc	fc	fc			
AP105	280	ff	ff	ff	ff	fc			
AP105	281	fc	fc	fc	fc	fc			
AP105	282	ff	fc	fc	fc	fc			
AP105	283	fc	fc	fc	fc	fc			
AP105	284	ff	fc	fc	fc	fc			
AP105	285	fc	fc	fc	fc	fc			
AP105	286	fc	fc	fc	fc	fc			
LB132	1						fc	fc	
LB132	2						fc	fc	
LB132	3						ff	ff	Fuse
LB132	4						ff	ff	Fuse
LB132	5						ff	ff	Fuse
LB132	6						fc	fc	
LB132	7						ff	fc	
LB132	8						ff	ff	Fuse

LB132	9	ff	ff	Fuse
LB132	10		fc	
LB132	11	ff	ff	Fuse
LB132	12	ff	ff	Fuse
LB132	13	ff	ff	
LB132	14	ff	ff	
LB132	15	fc	fc	
LB132	16	fc	ff	
LB132	17	ff	fc	
LB132	18	ff	ff	
LB132	19	fc	fc	
LB132	20	fc	fc	
LB132	21	ff	ff	
LB132	22	ff	fc	
LB132	23	fc	fc	
LB132	24		fc	
LB132	25			
LB132	26	fc	fc	
LB132	27	fc	fc	
LB132	28	ff	ff	
LB132	29	fc	fc	
LB132	30	ff	ff	
LB132	31	fc	ff	
LB132	32	ff	fc	
LB132	33	ff	ff	
LB132	34		fc	
LB132	35	fc	fc	
LB132	36	fc	fc	
LB132	37	ff	fc	
LB132	38	fc	ff	
LB132	39	fc	fc	
LB132	40	ff	ff	
LB132	41	fc	fc	
LB132	42	ff	ff	
LB132	43	fc	fc	
LB132	44	fc	fc	
LB132	45	ff	ff	
LB132	46	ff	ff	
LB132	47	fc	fc	
LB132	48	fc	fc	
LB132	49	ff	ff	

LB132	50		ff	ff									
LB132	51		fc	fc									
LB132	52		fc	fc									
LB132	53		ff	ff									
LB132	54		ff	ff									
LB132	55		ff	ff									
LB132	56		fc	fc									
LB132	57		fc	fc									
LB132	58		ff	ff									
LB132	59		ff	ff									
LB132	60		fc	fc									
LB132	61		fc	ff									
LB132	62		ff	ff									
LB132	63		ff	ff									
LB132	64		fc	fc									
LB132	65		fc	fc									
LB132	66		ff	fc									
LB132	67		fc	fc									
LB132	68		ff	ff									
LB132	69		fc	fc									
LB132	70		fc	fc									
LB132	71		ff	fc									
LB125	5	rr			rr	rr				Reject	Reject	Fuse	Fuse
LB125	7	rr			rr	rr				Reject			
LB125	11	rr			rr	rr				Reject		Fuse	Fuse
LB125	22	rr			rr	rr					Reject	Fuse	Fuse
LB125	25	rr			rr	rr					Reject		Fuse
LB125	27	rr			rr	rr				Reject	Reject	Fuse	Fuse
LB125	30	rr			rr	rr					Reject	Fuse	
LB125	31	rr			rr	rr					Reject	Fuse	Fuse
LB125	41	rr			rr	rr					Reject		
LB125	42	rr			rr	rr							Fuse
LB140	27						ff	ff			Fuse		Reject
LB140	55						ff	ff			Fuse		Reject
LB223	2						ff	ff	Fuse				
LB223	8						ff	ff	Fuse				
LB223	9						ff	ff	Fuse				
LB230	9						ff	ff	Fuse				
LB230	11						ff	ff	Fuse				
LB230	14						ff	ff	Fuse				
LB232	1						ff	ff	Fuse				

[1] Raw data for ARC-fr used for comparison in figure 3 previously published and available at http://www.genetics.org/content/vol0/issue2007/images/data/genetics.107.075689/DC1/genetics.107.075689-2.txt

[2] Crosses as designated in Table S2

[3] ARC haplotype, Markers as listed in Table S1

[4] ARC-ff testers: 217-11, 833-8, AP100-88; ARC-rr testers: 4117-2, MP104-34
